# Application of Multivariate Statistical Analysis to Simultaneous Spectrophotometric Enzymatic Determination of Glucose and Cholesterol in Serum Samples

**DOI:** 10.1155/2019/7532687

**Published:** 2019-01-02

**Authors:** Jessica Torres-Gamez, Jose A. Rodriguez, M. Elena Paez-Hernandez, Carlos A. Galan-Vidal

**Affiliations:** Universidad Autonoma del Estado de Hidalgo, Area Academica de Quimica, Carr. Pachuca-Tulancingo Km. 4.5, 42184 Mineral de la Reforma, HGO, Mexico

## Abstract

A method using UV-Vis spectroscopy and multivariate tools for simultaneous determination of glucose and cholesterol was developed in this paper. The method is based on the development of the reaction between the analytes (cholesterol and glucose) and enzymatic reagents. The spectra were analyzed by partial least squares regression and artificial neural networks. The precision estimated between nominal and calculate concentration demonstrate that artificial neural network model was adequate to quantify both analytes in serum samples, since the % relative error obtained was in the interval from 5.1 to 8.3. The proposed model was applied to analyze blood serum samples, and the results are similar compared to those obtained employing the reference method.

## 1. Introduction

Glucose and cholesterol play significant roles in a series of human physiological processes. It is well recognized that blood glucose of a high level is the most typical warning of diabetes. According to World Health Organization, approximately 3.4 million people died from high blood sugar in 2004 and diabetes will be 7th leading cause of death in 2030 [1]. An elevated level of blood cholesterol increased the risk of cardiac and brain vascular diseases such a hypertension, arteriosclerosis, coronary heart disease, lipid metabolism dysfunction, and brain thrombosis. High cholesterol level has also been implicated in nephrosis, diabetes, jaundice, and cancer [2]. Overall, an increased cholesterol level is estimated to cause 2.6 million deaths and 29.7 million disability adjusted life years [1–3].

Nowadays, a variety of methods have been developed for blood glucose or cholesterol assays such as amperometry, electrochemical method, mass spectrometric, near-infrared spectra, and high performance liquid chromatography [3]. However, these techniques require expensive instruments, advanced analytical skills, and considerable amount of time. For general purposes, it is important to develop rapid and robust methods that are directly applicable or simply pretreated samples. UV-Vis spectrometry is widely employed to determine glucose and cholesterol in clinical samples as a reference method [4].

Glucose is determined using a colorimetric enzymatic assay with glucose oxidase to produce hydrogen peroxide. Phenol reacts with 4-aminophenazone and the product form is oxidized by hydrogen peroxide to generate a chromophore quinone-imine compound which absorbance is proportional to the original concentration of glucose in the serum sample [4]. Cholesterol is determined by a colorimetric enzymatic assay with cholesterol esterase and cholesterol oxidase to generate 4-cholesten-3-one and hydrogen peroxide, followed by the same reaction described for glucose [5]. Despite both methodologies are based on the analysis of the same oxidizing product, the analytical sensitivities are different. Therefore, the development of an effective method for simultaneous determination of glucose and cholesterol can be possible.

Quantitative UV-Vis methodologies employing chemometric techniques have particularly attracted attention due to the possibility of analyzing simultaneously two or more analytes. The most commonly used chemometric tools are multilinear regression (MLR), partial least squares regression (PLS), and artificial neural networks (ANN). ANN is a data processing system consists of a large number of highly interconnected elements in an architecture inspired by the structure of brain, and it is a powerful modeling tool for processing complex or imprecise data [6, 7].

The applications of spectrometry combined with chemometric methods to the determination of multicomponent substance have been reported for analysis of mixtures of sugars, flavonoids, compounds in cold medicines, dipyrone, and papaverine in pharmaceutical formulation [6, 8–10]. This paper presents a UV-Vis spectrometry study combined with chemometric tools for simultaneous determination of glucose and cholesterol in serum samples.

## 2. Materials and Methods

### 2.1. Chemicals

Cholesterol enzymatic reagent was composed of cholesterol esterase (CHE, 1000 U/L), cholesterol oxidase (CHOD, 300 U/L), peroxidase (POD, 650 U/L) phenol, and 4-aminophenazone (4-AP). Glucose enzymatic reagent was composed of glucose oxidase (GOD, 15000 U/L), peroxidase (POD, 1000 U/L), phenol, and 4-AP. Glucose aqueous primary standard (100 mg/dL), cholesterol aqueous primary standard (200 mg/dL), enzymatic reagents, and primary standards were supplied by Spinreact (Girona, Spain). Triton X-114 and 3-[N-Morpholino] propanesulfonic acid (MOPS, 99.5%) were purchased from Sigma-Aldrich (Sigma, St. Louis, MO, USA). MOPS buffer solution (10 mM, pH 6) was prepared by using MOPS and Triton X-114 (10% w/v). All solutions were prepared in deionized water obtained in a Mili-Q water purification system (Milipore, Bedford, MA, USA).

### 2.2. Sample Analysis

Serum was prepared by centrifugation at a speed of 1500 rpm for 20 min. Serum samples were collected from volunteers in 6 mL red vacutainer tubes. All samples were frozen at -4°C until their analysis [11].

Spectrometric measurements were performed using Perkin Elmer Lambda 40 UV-Vis spectrophotometer (Perkin Elmer, Madrid, Spain). UV-Vis spectrum was recorded between 380 nm and 800 nm (at 5 nm intervals). Absorbance spectra of samples were obtained employing quartz cell (10 mm light path, Hellma Analytics, mod. 104-10-40). After each measurement, the cell was cleaned using deionized water. Experimental data were smoothed by Savitzky-Golay method with five-point window [12].

### 2.3. Multivariate Determination

Stock solutions of glucose (100 mg/L) and cholesterol (100 mg/L) were prepared from primary standard in MOPS buffer. Standard solutions (0.2-0.6 mg/dL) for simultaneous study were prepared by mixing aliquots of different concentrations of glucose and cholesterol employing a 32-concentration matrix. Mixtures were including 1 mL of MOPS buffer, 1 mL of glucose enzymatic reagent, and 1 mL of cholesterol enzymatic reagent and deionized water until completing 10 mL. For serum samples analysis, 20 *μ*l aliquot was taken, following the same procedure described for mixture solutions.

For data analysis, the region of 400–600 nm (each 5 nm, 41 data each spectra) was selected because this contains suitable spectral information from the interesting component mixture. The samples were divided into calibration (13 samples, matrix 13×41) and prediction (9 samples, matrix 9×41) sets. PLS was done employing Minitab version 17.1 (Minitab Inc., State College, PA, USA). ANN Data analysis was performed using MATLAB version 9.2 (The Math-Works, Natick, USA) using the NN-toolbox.

### 2.4. Reference Method

Serum glucose and cholesterol levels were measured individually using previously described methodologies employing the kits supplied by Spinreact. Samples were treated and analyzed following the instructions provided by maker. Serum solutions were prepared with 10 *μ*L of sample and 1 mL of glucose or cholesterol reagent, as the case may be. Blank solution was including 1 mL of reagent and standard solution was including 10 *μ*L of primary standard and 1 mL of reagent. All solutions were analyzed by UV-Vis spectrophotometry.

## 3. Results and Discussion

**Figure 1:**
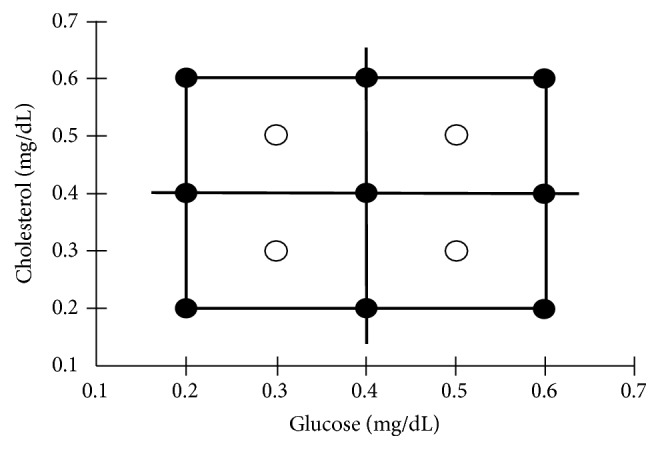
Factorial design. (●) Experiments of design. (○) Internal validation points.

The composition of the calibration set was constructed according to factorial design (3^2^) + 4 internal validation points (Figure 1). The scanning of these solutions was monitored at the wavelengths ranging mentioned in sample analysis section.

**Figure 2:**
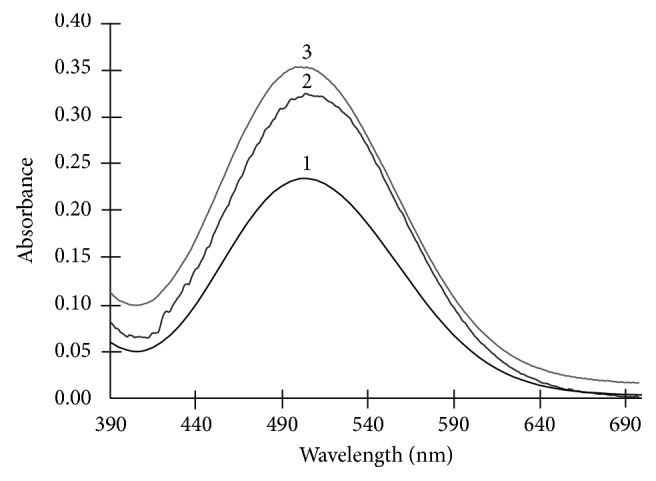
UV-Vis spectra of (1) mixture of glucose (0.6 mg/dL) and cholesterol (0.6 mg/dL), (2) 2.5 mg/dL of glucose, and (3) 2.5 mg/ dL of cholesterol.

The spectral analysis suggested that the maximum amounts of glucose and cholesterol absorption were at 505 nm, for both analytes (Figure 2). The maximum absorption obtained is congruent to the information provided in the enzyme kit.

Absorbance* versus* concentration relationship of the 13 combined solutions (glucose + cholesterol) was analyzed by PLS. Root-mean-square error of validation (RMSEC), root-mean-square error cross validation (RMSECV), predicted residual error sum squares (PRESS), correlation coefficient for calibration, and cross validation were employed to select the adequate model. Acceptable models should have low RMSEC and RMSECV and high correlation coefficients. Besides, the differences between RMSECV and PRESS should be small [13, 14]. The parameters of the PLS calibration models are shown in Table 1. As can be seen, the R2 was high with the smoothed data set, the RMSEC was low in both cases, and the difference between RMSECV and PRESS was higher in the first data set than in the pretreated data set. Although, the pretreated data set presented better results in RMSEC and RMSECV; the difference between RMSECV and PRESS was high, which shows that there is dispersion among the data.

To check the robustness of the PLS calibration models, the models were applied to an independent prediction set (internal validation points), which was different from those upon which the calibration model was built. Results are presented in Table 2. The values of relative errors of predictions (%REP) were in the range between 27% and 64%; this indicates that the application of the PLS model shows an inadequate predictive ability for the simultaneous quantification of glucose and cholesterol.

Based on these results, we applied the ANNs methodology to determine the concentration of glucose and cholesterol in serum sample. In order to select the best neural network, different architectures were compared to build and validate the predictive model of the network, which consisted of an input layer, one hidden, and one output. The number of neurons of the input layer was equal to the number of independents variables entered into the model; in this case 41 data are obtained from the absorbance in the range of 400-600 nm (measurement 5 nm). The number of neurons in the output layer corresponded to the number of model output variables; in this study is concentration of glucose and cholesterol.

On the other hand, the number of neurons in the hidden layer was obtained from the best architecture of ANNs through the following procedure: ANNs with a number “N” of neurons in the hidden layer was created and the type of training and the transfer function was defined. The network was trained with the calibration data set, considering 70% of learning, a 15% for monitoring, and 15% to test the network. The above procedure was made by combining different types of training and transfer functions. After obtaining the errors of different architectures, we selected the best amount of the hidden layer neurons, the transfer function, and the training type based on the least mean squared normalized error (MSE), REP%, and RMSE (mg/dL) for two analytes [15].

Therefore, the procedure described to find the most suitable network architecture for the resolution of measured signals was applied. Thus, the best network model was obtained using Tansig (tangent sigmoid) transfer function in the hidden layer and the purelin linear function for the output layer. The most appropriate algorithm in the training stage was that of Levenberg-Marquardt [16]. Optimized parameters and error estimated from ANNs methodology are shown in Table 3. The procedure was repeated with data set (smoothing 5 points); results are presented in Table 4.

After optimizing the ANNs with the set of calibration, a separate set (internal validation points) was used to perform the validation prediction, through the analysis of accuracy and precision. Results obtained from the validation set are shown in Table 5 for each analyte, respectively. The lowest RMSEP values corresponded to relative errors of prediction in the range between 5% and 8%, good enough to accept the method as accurate [7], while the REP values obtained by applying PLS method were above 50% (Table 2).

On the other hand, the proposed ANN method was also applied to the determination of glucose and cholesterol in serum samples.  Table 6 lists the values of glucose and cholesterol obtained by using the proposed method and the corresponding values reported by reference method. As we can observe, the two types of results are comparable even though there are few differences, indicating that the ANN method could be used in simultaneous determination of glucose and cholesterol.

## 4. Conclusions

The method of UV-Vis spectrometry combined with chemometrics tools for simultaneous determination of glucose and cholesterol was established in this paper. It was found that ANN was the most appropriated method to predict the values of analytes. Comparing the results with the reference method, the method developed in this work presents no significant differences. The proposed (UV-Vis ANN) method is rapid and relatively inexpensive and it facilitates simultaneous determination of glucose and cholesterol in serum samples.

## Figures and Tables

**Table 1:** Parameters of PLS model (400-600 nm, 5 nm interval).

	Analytes	Calibration		Cross-validation		
Model		R^2^	RMSEC (mg/dL)	R^2^	RMSECV (mg/dL)	PRESS
PLS	Glucose	0.9469	0.0376	0.5931	0.1041	0.9764
	Cholesterol	0.9222	0.0455	0.1594	0.1497	2.0174

PLS, smoothing 5 pts	Glucose	0.9483	0.0383	0.5633	0.1076	1.0499
	Cholesterol	0.9252	0.0446	0.3739	0.1292	1.5025

**Table 2:** Errors obtained between nominal concentrations and estimated by PLS.

Model	Analytes	RMSEP^1^ (mg/dL)	%REP
PLS	Glucose	0.1263	27.8
	Cholesterol	0.3192	64.5

PLS, smoothing 5 pts	Glucose	0.1212	28.9
	Cholesterol	0.3105	64.9

^1^Root mean square error of prediction.

**Table 3:** Optimized parameters and errors obtained by ANNs.

	Glucose	Cholesterol
Architecture	41-6-1	41-10-1
Number of iterations	20	24
Hidden layer transfer function	Tansig	Tansig
RMSETr^1^ (mg/dL)	0.0241	0.0720
RMSEM^1^ (mg/dL)	0.1221	0.1130
RMSET^1^ (mg/dL)	0.0730	0.3809
RMSE, P^2^ (mg/dL)	0.3865	0.5104
REP^2^ (%)	9.66	12.76

^1^They are the RMSE for training, monitoring, and testing for the calibration set, respectively.

^2^Errors obtained between nominal and estimated concentrations by ANNs for the calibration set.

**Table 4:** Parameters and errors obtained with data set (smoothing 5 pts) by ANNs model.

	Glucose	Cholesterol
Architecture	41-5-1	41-5-1
Number of iterations	14	24
Hidden layer transfer function	Tansig	Tansig
RMSET (mg/dL)	0.0079	0.0618
RMSEM (mg/dL)	0.0621	0.1990
RMSET (mg/dL)	0.1154	0.1077
RMSE, P (mg/dL)	0.2948	0.5180
REP (%)	7.37	12.94

**Table 5:** Errors obtained between nominal concentrations and estimated by ANN.

Model	Analytes	RMSEP (mg/dL)	%REP
ANN	Glucose	0.0221	5.5
	Cholesterol	0.0303	7.6

ANN, smoothing 5 pts.	Glucose	0.0211	5.3
	Cholesterol	0.0324	8.1

**Table 6:** Application of the ANN method for the estimation of glucose and cholesterol in serum samples.

Reference method		Found value (ANN)				Found value (ANN, smoothing 5 pts.)			
Glc^1^ (mg/dL)	TC^2^ (mg/dL)	Glc (mg/dL)	%RE^3^	TC (mg/dL)	%RE	Glc (mg/dL)	%RE	TC (mg/dL)	%RE
87	245	79.5	8.6	246.2	0.5	78.0	10.4	225.1	8.1
102	193	106.7	4.6	187.5	2.8	95.6	6.3	180.0	6.7
102	289	95.1	6.8	279.8	3.2	93.9	7.9	269.3	6.8
102	200	105.6	3.5	201.7	0.9	94.4	7.4	188.4	5.8
103	239	100.3	2.6	224.5	6.1	107.6	4.5	224.6	6.0
108	213	113.6	5.2	211.8	0.6	106.7	1.2	224.3	5.3
83	139	81.6	1.7	139.9	0.6	88.2	6.3	134.4	3.3
100	80	104.7	4.7	81.7	2.1	103.8	3.8	84.6	5.8
72	75	79.7	10.7	78.7	4.9	66.5	7.7	77.0	2.7

^1^Glc: glucose, ^2^TC: total cholesterol, and ^3^%RE: relative error.

## Data Availability

The data used to support the findings of this study are available from the corresponding author upon request.
